# Direct visualization of a significant stenosis of the right coronary artery by transthoracic echocardiography. A case report

**DOI:** 10.1186/1476-7120-5-33

**Published:** 2007-10-01

**Authors:** Espen Holte, Johnny Vegsundvåg, Rune Wiseth

**Affiliations:** 1 Department of Internal Medicine, Ålesund Hospital, Ålesund, Norway; 2 Department of Circulation and Medical Imaging, Norwegian University of Science and Technology, Trondheim, Norway; 3 Department of Cardiology, Trondheim University Hospital, Trondheim, Norway

## Abstract

Non-invasive imaging of coronary arteries by transthoracic echocardiography is an emerging diagnostic tool to study the left main (LM), left descending artery (LAD), circumflex (Cx) and right coronary artery (RCA). Impaired coronary circulation can be assessed by measuring coronary velocity flow reserve (CVFR) by transthoracic Doppler echocardiography. Coronary artery stenoses can be identified as localized colour aliasing and accelerated flow velocities. We report a case with an acute coronary syndrome (ACS) of a 46-year-old man. With non-invasive imaging of coronary arteries by transthoracic echocardiography (TTE), we identified a segment of the mid right coronary artery (RCA) suggestive of stenosis with localized colour aliasing and accelerated flow velocity. We found a high ratio between the stenotic peak velocity and the prestenotic peak velocity, and a pathologic coronary flow velocity reserve (CFVR) distal to the stenosis in the posterior interventricular descending branch (RDP). Subsequent coronary angiography demonstrated one vessel disease with a stenosis in segment 3 of RCA, which was successfully treated with percutaneos coronary intervention PCI. Two weeks following the PCI procedure he was readmitted to hospital with chest pain. A subacute stent thrombosis was questioned, and repeated echocardiography was preformed. The mid portion of RCA showed normal and laminar flow. The CVFR of RCA measured in the RDP showed normal vasodilatory response, confirming an open RCA without any flow limitation. A repeated coronary angiogram demonstrated only a mild in stent intimal hyperplasia. This case illustrates the value of transthoracic echocardiography as a tool both in the diagnosis and the follow-up of chest pain disorders and coronary flow problems. Transthoracic echocardiography allows both direct visualization of the various coronary segments and assessment of the CVFR.

## Background

Non-invasive imaging of coronary arteries by transthoracic echocardiography is an emerging diagnostic tool to study the left main (LM), left descending artery (LAD), circumflex (Cx) and right coronary artery (RCA) [1]. Impaired coronary circulation can be assessed by measuring coronary velocity flow reserve (CVFR) by transthoracic Doppler echocardiography [2-4]. Coronary artery stenoses can be identified as localized colour aliasing and accelerated flow velocities [5,6]. We report a case with an acute coronary syndrome (ACS), where transthoracic echocardiography showed a significant stenosis of the middle part of the RCA, confirmed by pathologic CVFR measured in the posterior interventricular descending branch (RDP) and by coronary angiography. The stenosis was successfully treated by percutaneos coronary intervention (PCI) with drug eluting stent. The patient was readmitted to hospital 2 weeks later after a new episode of chest pain. Transthoracic echocardiography did not indicate stent related flow reduction with normal CVFR of the RDP. Coronary angiography performed 3 months later demonstrated minor in stent intimal hyperplasia with no significant stenosis.

## Case presentation

A 46-year-old man presented with typical retrosternal chest pain compatible with ACS. ECG was normal while cardiac troponin I was slightly elevated at 0.06 μcg/L(≤ 0.04 μcg/L as normal reference). He was treated with acetylsalicylic acid, clopidogrel, atorvastatin, carvedilol, and subcutaneous enoxaparin. There were no episodes of recurrent chest pain.

The patient was admitted to our hospital during a pilot phase of a study comparing transthoracic coronary flow measurements by ultrasound with results obtained at coronary angiography. Accordingly, the patient was examined with an Acuson Sequoia c 512 (Siemens Medical Solutions USA, Inc) ultrasound system connected to a standard 3.5 MHz transthoracic transducer. Left ventricular contractility was normal. With the use of all standard and modified apical, parasternal and subcostal views [5], we identified by colour flow Doppler the LM, the proximal, mid and distal segment of the LAD, the proximal and mid segment of the Cx, the proximal, mid and distal segments of the RCA, and the RDP. The velocity scale was set to 0.24 m/s, but was actively changed. Angle correction was used. Contrast agent was not used.

**Figure 1:**
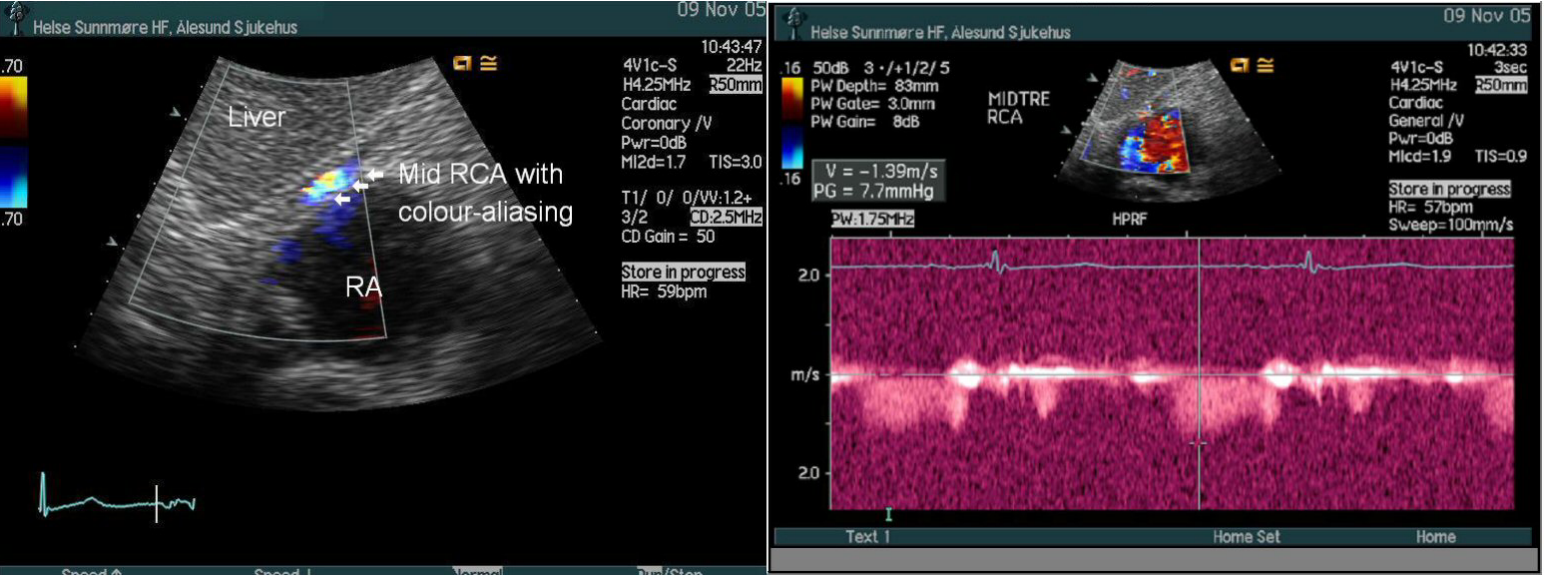
In the modified subcostal four-chamber window we identified a segment of the mid RCA with colour-aliasing suggestive for a stenosis. Stenotic diastolic peak velocity measured with pulsed wave Doppler 1.39 m/s, in a modified subcostal four chamber view.

The LM, LAD, proximal and mid segments of the Cx, RDP and proximal segment of RCA showed laminar flow and normal flow velocities, without any signs of colour-aliasing suggestive of stenosis. In the subcostal 4-chamber view we identified a segment of the mid RCA with colour-aliasing (Figure 1), and the diastolic peak velocity measured with pulsed wave Doppler was 1.39 m/s (Figure 1). These findings were confirmed with the use of a modified subcostal short axis view. The flow acceleration was quantified as the ratio of peak diastolic flow velocity at the site of aliasing to nearest upstream non-accelerated prestenotic peak diastolic flow velocity. This ratio was 3.75 (Table 1), compatible with a significant stenosis in the mid RCA.

**Table 1:** Measurements

1	SPDV: 1.39 m/s	PPDV: 0.37 m/s	Ratio_SPDV/PPDV _= 3.75
	Flow velocity at baseline	Flow velocity at adenosine	Ratio_adenosine/baseline_
2	0.28 m/s	0.78 m/s	CFVR_LAD _= 2.78
3	0.41 m/s	0.48 m/s	CFVR_RDP _= 1.17
4	0.38 m/s	0.83 m/s	CFVR_RDP _= 2.18

1: Ratio between the stenotic peak velocity (SPDV) and the prestenotic peak velocity (PPDV)

2: Coronary flow velocity reserve of the LAD

3: Coronary flow velocity reserve of the RDP before PCI

4: Coronary flow velocity reserve of the RDP at readmission

**Figure 2:**
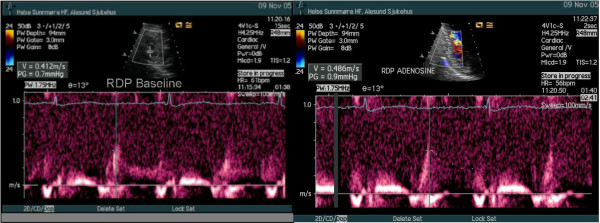
CVFR of RDP. Peak diastolic velocity of RDP baseline 0.41 m/s. Peak diastolic velocity of RDP adenosine (enveloped) 0.48 m/s.

From a modified apical 2-chamber view the CVFR was measured both in the RDP and the distal segment of LAD, and hyperaemic flow was obtained by venous infusion of adenosine (140 μcg/kg/min). The CVFR of the LAD was normal (Table 1), indicating that the LAD was open with good microvascular function and without any significant stenosis. The CVFR value of 1.17 measured in the RDP was clearly pathologic (Table 1, Figure 2), indicating an obstructive lesion proximal to the RDP, most likely in the mid segment of the RCA.

**Figure 3:**
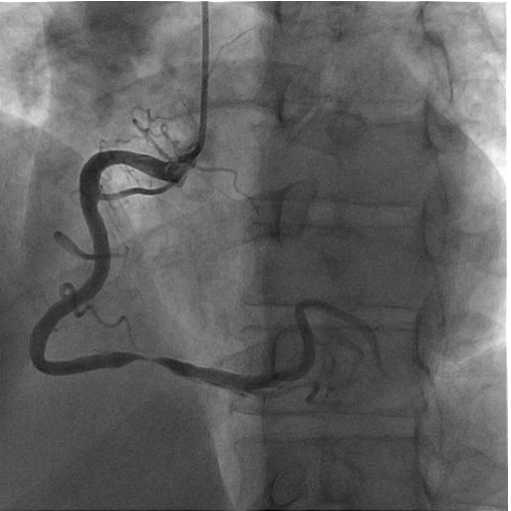
Angiography shows the stenosis of RCA.

**Figure 4:**
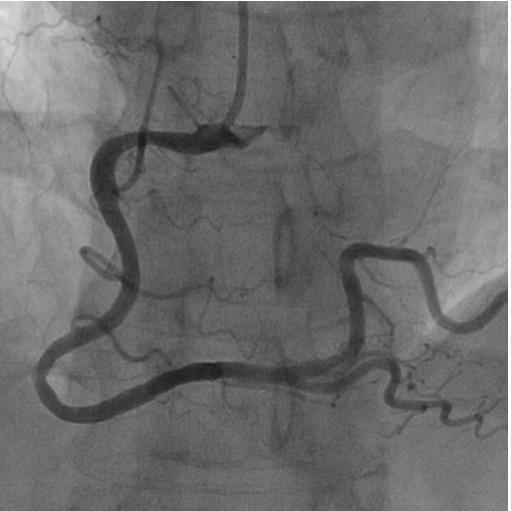
Angiography shows RCA after PCI.

Subsequent coronary angiography demonstrated one vessel disease with a stenosis in segment 3 of the right coronary artery (Figure 3). The location of the stenosis correlated well with our echocardiographic description. The stenosis was successfully treated with PCI and implantation of a drug eluting stent (Figure 4).

**Figure 5:**
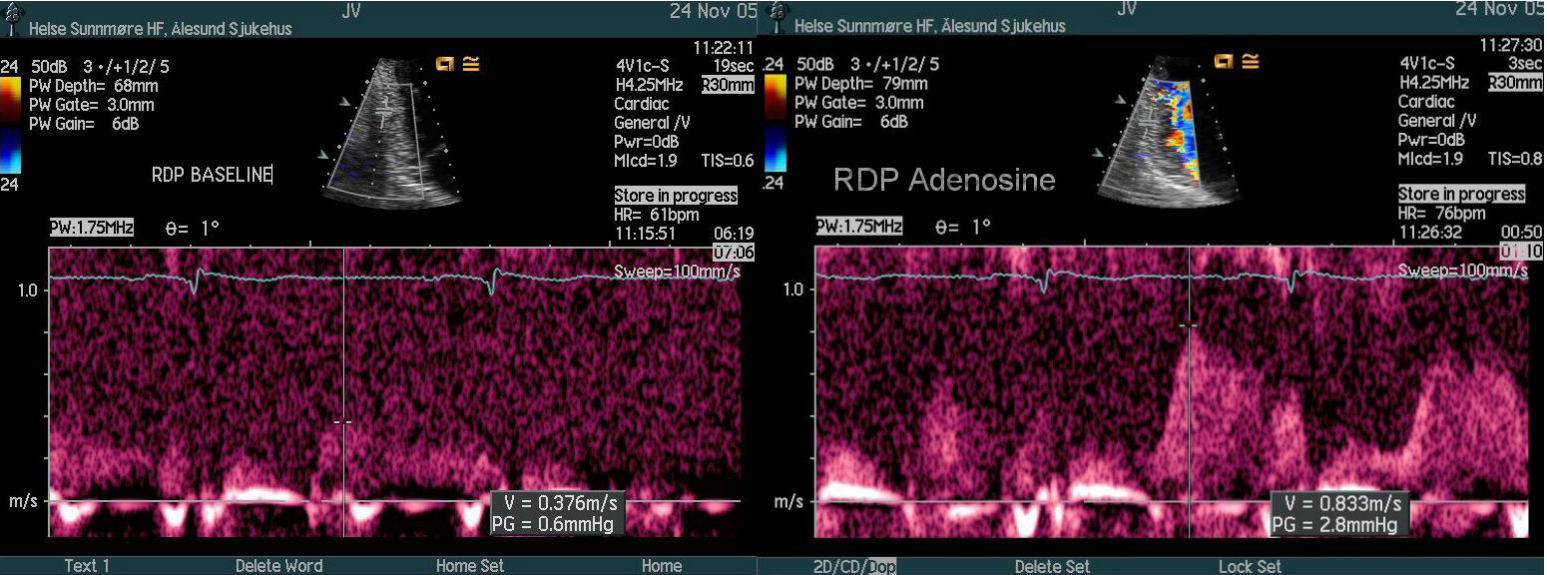
CVFR of RDP after PCI by readmission. Peak diastolic velocity of RDP baseline 0.38 m/s. Peak diastolic velocity of RDP adenosine 0.83 m/s.

Two weeks following the PCI procedure the patient was readmitted to hospital with chest pain. ECG and level of cardiac troponin I was normal. A subacute stent thrombosis was questioned, and a repeated echocardiography was performed. The mid portion of the RCA was thoroughly scanned, with findings of normal and laminar flow without any colour flow aliasing, suggestive of no flow impairment at the stented segment. Repeated CVFR of the RDP showed a normal vasodilatory response (Table 1, Figure 5), confirming an open RCA without any flow limitation. A more thorough clinical assessment concluded with thoracic myalgia as the probable cause of his recent chest pain. Nevertheless, a repeated coronary angiogram was performed three months later demonstrating only mild in stent intimal hyperplasia.

## Discussion

Several recent reports have documented the feasibility of visualizing most segments of the main coronary arteries by transthoracic echocardiography [1,5]. By this technique, coronary stenoses typically show local flow acceleration and turbulence expressed as colour aliasing by colour flow Doppler and accelerated flow velocities across the stenosis [5,6]. To assess the severity of the stenoses, flow acceleration can be quantified by comparing flow velocities at the site of aliasing with nearest upstream non-accelerated prestenotic flow velocities [5,7]. A maximal-to-prestenotic peak diastolic flow velocity ratio greater than 2.0 predicts significant stenosis with high sensitivity and specificity [5]. Our patient demonstrated in the mid RCA a significantly accelerated flow velocity of 1.39 m/s (Figure 1) and an elevated maximal-to-prestenotic ratio of 3.75 (Table 1), compatible with a significant stenosis in this segment. This was confirmed by coronary angiography (Figure 3). A comprehensive examination of the coronary arteries by transthoracic echocardiography did not reveal other stenoses, as confirmed by coronary angiography.

In normal coronary arteries without an epicardial flow-limiting stenosis, CVFR is controlled by the microcirculatory resistances, and coronary hyperemia can be induced by vasodilatory drugs and physiologic stimuli decreasing the resistances of microcirculation. Hemodynamic significant epicardial coronary stenoses create strong proximal resistances that are higher than that opposed from the microcirculation, and reduce the CVFR [8]. Several recent reports have documented the feasibitity of transthoracic echocardiography (with the pulsed Doppler sample volume placed distally to the flow-limiting stenosis) of estimating the CVFR [2-4,8]. A CFVR value of less than 2 has been widely adopted to indicate one or more hemodynamic significant stenoses located more proximally in the coronary artery [8], documented for the LAD [4] and RDP [3]. Our patient showed pathologic CVFR of the RDP (Table 1, Figure 2) and normal CVFR of the LAD (Table 1), confirming the findings of a significant stenosis in the RCA and an open LAD.

With the use of transthoracic echocardiography, both direct visualization of formerly angioplasty-treated segments [9] and CVFR measurements [10] have been reported as suitable methods of follow-up of treated stenoses. After a successful PCI treatment of the stenosis in the mid RCA, our patient presented with chest pain suggestive of possible restenosis. However, both direct visualization of this segment and CVFR measurement of the RDP (Table 1, Figure 5) indicated normal flow in the RCA as was confirmed by coronary angiography.

## Conclusion

This case illustrates the value of transthoracic echocardiography as a tool both in the diagnosis and the follow-up of chest pain disorders and coronary flow problems. Transthoracic echocardiography allows both direct visualization of the various coronary segments and assessment of the CVFR of the LAD and RCA. With the use of these techniques, we were able to correctly identify a hemodynamic significant stenosis of the mid RCA. When the patient was readmitted with chest pain we could exclude flow limitation in the stented segment by transthoracic ultrasound examination of the RCA.

## References

[B1] Krzanowski M, Bodzoń W, Dimitrow PP (2003). Imaging of all three coronary arteries by artery transthoracic echocardiography. An illustrated guide. Cardiovascular Ultrasound.

[B2] Hozumi T, Yoshida K, Akasaka T, Asami Y, Ogata Y, Takagi T, Kaji S, Kawamoto T, Ueda Y, Morioka S (1998). Noninvasive assessment of coronary flow velocity and coronary flow velocity reserve in the left anterior descending artery by Doppler echocardiography. Comparison with invasive technique. J Am Coll Cardiol.

[B3] Tokai K, Watanabe H, Hirata K, Otsuka R, Muro T, Yamagishi H, Yoshiyama M, Hozumi T, Yoshikawa J (2003). Noninvasive assessment of myocardial ischemia in the left ventricular inferior regions by coronary flow reserve measurement using transthoracic Doppler echocardiography. J Am Soc Echocardiogr.

[B4] Matsumura Y, Hozumi T, Watanabe H, Fujimoto K, Sugioka K, Takemoto Y, Shimada K, Muro T, Yoshiyama M, Takeuchi K, Yoshikawa J (2003). Cut-off value of coronary flow velocity reserve by transthoracic Doppler echocardiography for diagnosis of significant left anterior descending artery stenosis in patients with coronary risk factors. Am J Cardiol.

[B5] Saraste M, Vesalainen RK, Ylitalo A, Saraste A, Koskenvuo JW, Toikka JO, Vaittinen MA, Hartiala JJ, Airaksinen KE (2005). Transthoracic Doppler echocardiography as a noninvasive tool to assess coronary artery stenoses – a comparison with quantitative coronary angiography. J Am Soc Echocardiogr.

[B6] Krzanowski M, Bodzoń W, Brzostek T, Niżankowski R, Szczeklik A (2000). Value of transthoracic echocardiography for the detection of high-grade coronary artery stenosis: Prospective evaluation in 50 consecutive patients scheduled for coronary angiography. J Am Soc Echocardiogr.

[B7] Hozumi T, Yoshida K, Akasaka T, Asami Y, Kanzaki Y, Ueda Y, Yamamuro A, Takagi T, Yoshikawa J (2000). Value of acceleration flow and the prestenotic to stenotic coronary flow velocity ratio by transthoracic color Doppler echocardiography in noninvasive diagnosis of restenosis after percutaneous transluminal coronary angioplasty. J Am Coll Cardiol.

[B8] Rigo F (2005). Coronary flow reserve in stress-echo lab. From pathophysiologic toy to diagnostic tool. Cardiovasc Ultrasound.

[B9] Krzanowski M, Bodzoń W, Dudek D, Heba G, Rzeszutko M, Nizankowski R, Dubiel J, Szczeklik A (2004). Transthoracic, harmonic mode, contrast enhanced color Doppler echocardiography in detection of restenosis after percutaneous coronary interventions. Prospective evaluation verified by coronary angiography. Eur J Echocardiogr.

[B10] Lethen H, Tries HP, Brechtken J, Kersting S, Lambertz H (2003). Comparison of transthoracic echocardiography to intracoronary Doppler guidewire measurements for assessment of coronary flow reserve in the left anterior descending artery for detection of restenosis after coronary angioplasty. Am J Cardiol.

